# Energy recovery from human faeces via gasification: A thermodynamic equilibrium modelling approach

**DOI:** 10.1016/j.enconman.2016.04.005

**Published:** 2016-06-15

**Authors:** T. Onabanjo, K. Patchigolla, S.T. Wagland, B. Fidalgo, A. Kolios, E. McAdam, A. Parker, L. Williams, S. Tyrrel, E. Cartmell

**Affiliations:** Cranfield University, Cranfield, Bedfordshire MK43 0AL, United Kingdom

**Keywords:** Gasification, Exergy analysis, Biomass, Non-sewered sanitary systems, Nano Membrane Toilet

## Abstract

Non-sewered sanitary systems (NSS) are emerging as one of the solutions to poor sanitation because of the limitations of the conventional flush toilet. These new sanitary systems are expected to safely treat faecal waste and operate without external connections to a sewer, water supply or energy source. The Nano Membrane Toilet (NMT) is a unique domestic-scale sanitary solution currently being developed to treat human waste on-site. This toilet will employ a small-scale gasifier to convert human faeces into products of high energy value. This study investigated the suitability of human faeces as a feedstock for gasification. It quantified the recoverable exergy potential from human faeces and explored the optimal routes for thermal conversion, using a thermodynamic equilibrium model. Fresh human faeces were found to have approximately 70–82 wt.% moisture and 3–6 wt.% ash. Product gas resulting from a typical dry human faeces (0 wt.% moisture) had LHV and exergy values of 17.2 MJ/kg and 24 MJ/kg respectively at optimum equivalence ratio of 0.31, values that are comparable to wood biomass. For suitable conversion of moist faecal samples, near combustion operating conditions are required, if an external energy source is not supplied. This is however at 5% loss in the exergy value of the gas, provided both thermal heat and energy of the gas are recovered. This study shows that the maximum recoverable exergy potential from an average adult moist human faeces can be up to 15 MJ/kg, when the gasifier is operated at optimum equivalence ratio of 0.57, excluding heat losses, distribution or other losses that result from operational activities.

## Highlights

• On dry basis, typical human faeces contain 83 wt.% organic fraction and 17 wt.% ash.
• The LHV of dry human faeces ranged from 19 to 22 MJ/kg, values similar to wood biomass.
• Syngas from dry human faeces had LHV of 15–17 MJ/kg at equivalence ratio of ∼0.31.
• Energy is best recovered from moist human faeces at equivalence ratio above 0.6.
• Recoverable exergy potential from moist human faeces can be up to 15 MJ/kg.

## 1 Introduction

The development of sustainable energy systems is one of the top priorities for global development and the driving force for major political and societal reforms in the 21st century [13]. Despite the increasing world population and energy use, these energy systems are expected to provide technology solutions without: (i) competing with universal human needs, (ii) putting strain on the natural environment or, (iii) making unsustainable demands on limited available natural resources. There are propositions that the efficient use of energy, modification of existing energy-dependent and resource-intensive systems, and/or expansion of the use of renewable resources could encourage social, economic and environmental balance [42].

The flush toilet is a cost-, resource- and energy-intensive system. It demands the use of at least 3 L of water per flush [10] that can increase quickly up to 20 L, depending on toilet design and user’s behavioural pattern. It also requires sewer connections for transportation, treatment and disposal of excreta, wastewater and grey water. All these processes present energy requirements and the linear use of resources including chemicals are financial and environmental burdens for both urban and rural communities. Furthermore, the open discharge of sewage and residuals without treatment, gives rise to environmental and human health problems. Thus, the development of sustainable sanitary solutions requires a shift in the use of resources and energy paradigm.

The Reinvent the Toilet Challenge (RTTC) is one of the pioneering schemes initiated in 2011 under the Water, Sanitation & Hygiene (WASH) programme of the Bill and Melinda Gates Foundation to increase access to safe, sustainable and affordable sanitation [11]. The solutions developed under this initiative are expected to safely treat faecal waste, operate without water, sewer and external energy source, and provide business solutions with opportunities to recover useful resources such as energy, clean water and nutrients. The Nano Membrane Toilet (NMT) is a unique household-scale sanitary solution, being developed at Cranfield University. It is intended to treat human waste on-site without external supply of energy and water [27]. The proposed solution aims to significantly reduce the environmental and health impact of the poor sanitation, while minimizing the use of water, energy and land, which are main challenges and factors limiting the growth of developing and least developed countries. This toilet will deploy the use of a small-scale gasifier to convert the solid residues from human defecation into gas products of high energy value. The energy recovered from this process will be used to operate the toilet’s key components including the gasifier, thus ensuring a self-sustained system. Therefore, the optimal design of the NMT and similar sanitary solutions requires a good understanding of the recoverable exergy potential from human faeces and the optimal routes for thermal conversion.

Proper design and optimisation of the NMT system requires process modelling and integration. Thermodynamic equilibrium models are widely used to simulate the thermochemical conversion of fossil and renewable fuels. These models have been employed to examine the conversion of various feedstocks including refinery sludge, sewage sludge and manure [31], [2]. They provide insights into complex processes, allow the identification of critical parameters and optimum operating conditions, and facilitate process comparison [18], [12]. Their ability to predict product gas composition at given operating conditions have enabled an estimation of the energy recovery potentials from different biomass feedstocks [33], [30]. It has also influenced the development of alternate, improved and efficient process design and operation for biomass conversion [43], [35], [16]; hence, such models can provide insights for conceptual design of a gasifier for faeces conversion and product recovery schemes. The poor understanding of the suitability of human faeces as a feedstock for gasification is however a major limitation.

There is sparse information in the public domain on the properties of human faeces. Apart from the abundance of published literature in the medical sciences, with limited evidence for energy recovery analysis; there is little known on how faeces composition influence product and energy recovery. Although, gasification technology is widely applied for converting biomass feedstocks [20], most of the studies focused on faecal related materials typically utilise feedstocks such as animal manure, poultry waste, and at most sewage sludge [34], [17], [12]. Because sewage sludge is a by-product of industrial or municipal wastewater treatment processes and the composition varies significantly with source, time, space, and treatment methods, these studies cannot be directly applied to systems operating on human faeces. A few studies [19], [3] that have simulated the thermochemical conversion of human faeces have explored technologies such as plasma gasification, hydrothermal carbonization, with processes differing from the described study approach. More so, the experimental studies by Ward et al. [39], Muspratt et al. [26], Afolabi et al. [1] and Monhol and Martins [24], [23] have exploited technologies such as pyrolysis, hydrothermal carbonization and combustion to investigate product recovery from human faeces, and not gasification.

Thus, the aim of this study was to investigate the suitability of human faeces as a feedstock for gasification, as a first step for the development of an appropriate gasifier. It quantified the recoverable exergy potential from dry and moist human faeces and explored the optimal routes for thermal conversion of moist human faeces. The results were compared with the outputs from similar analysis on wood biomass and simulant faeces. A thermodynamic equilibrium model of the gasification scheme was developed in Aspen Plus. The effects of fuel characteristics and operating conditions including moisture content and equivalence ratio were examined on the gas product quantity and quality. Changes on the efficiency of the process were also evaluated.

## 2 Methods

### 2.1 Faecal biomass gasification process description

**Fig. 1:**
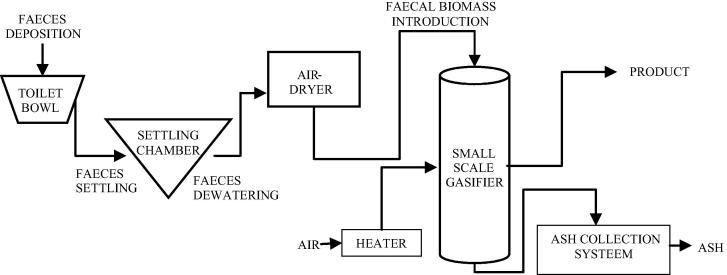
Flow process of the faecal biomass.

The process description of the human faeces from the point of deposition in the toilet to thermal conversion into gas is described in Fig. 1. The fresh human faeces is assumed to be partially dewatered following a settling process. The residual solids which still contain a high amount of moisture are then subjected to a drying stage, where part of the moisture is removed to an acceptable limit. The resultant biomass and an air stream are introduced into the small-scale-gasifier. The reactor allows sufficient time for the biomass to be converted to product gas with potential for recovery of thermal energy. The left-over ash is collected at the bottom of the unit.

### 2.2 Thermodynamic modelling of faecal biomass gasification

**Fig. 2:**
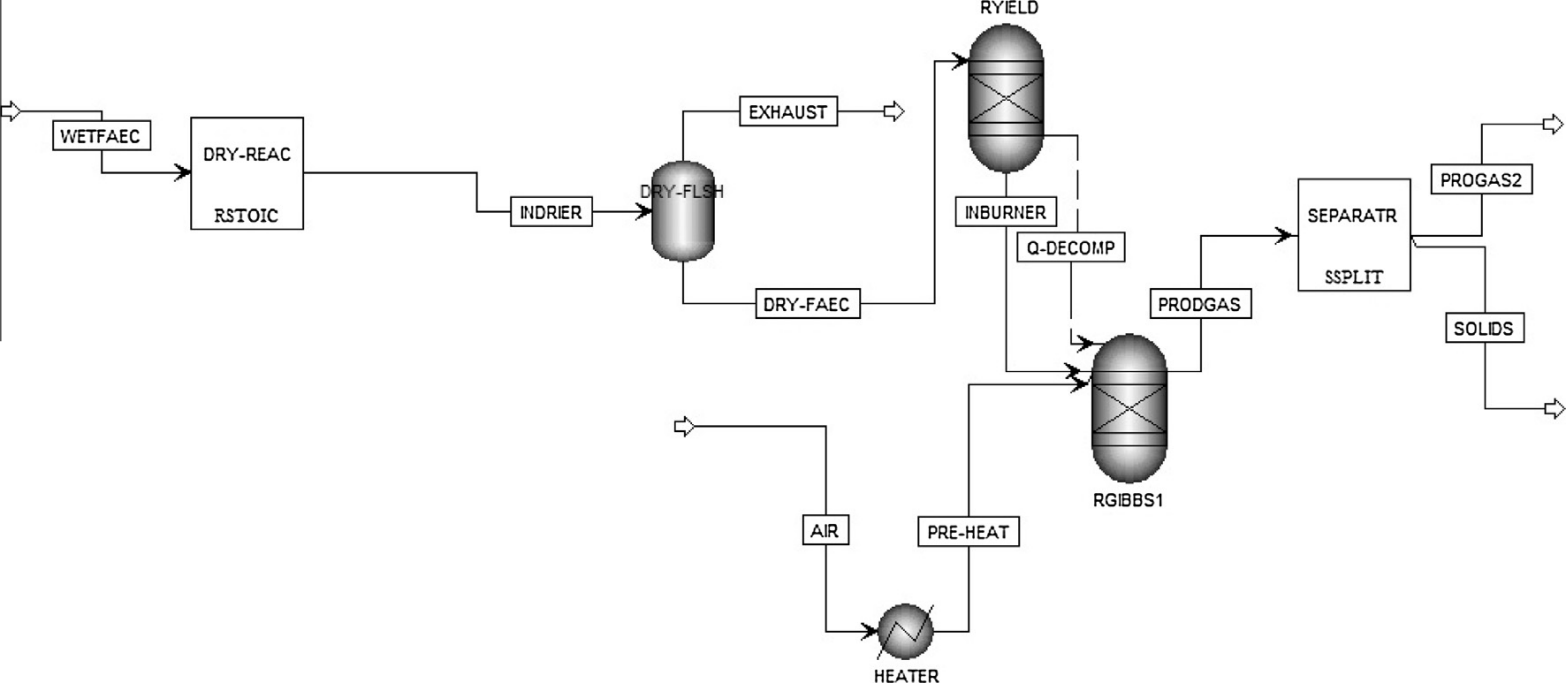
Schematic flow diagram as modelled on Aspen Plus for the gasification of faecal biomass.

The gasification of the faecal biomass as described in Fig. 1 was simulated in Aspen Plus using a non-stoichiometric chemical equilibrium based approach. The thermodynamic model was developed under the considerations of minimal energy requirement and for high recoverable heat and energy products. The dewatered biomass is introduced at standard temperature and pressure, then dried to an acceptable limit. Standard temperature and pressure air enters the process via a heater with capability of pre-heating the air, if required. The reaction between the biomass and the air stream leads to the conversion of the faecal biomass. In the presence of limited air, the product gas may include a combination of hydrogen (H_2_), carbon dioxide (CO), carbon monoxide (CO_2_), nitrogen (N_2_), water vapour or steam (H_2_O), methane (CH_4_), sulphur-containing compounds, other nitrogen-containing compounds, ash, and unconverted carbon. The composition of the gas products depends on the operating conditions (temperature and pressure) of the gasifier, fuel chemical composition and equivalence ratio. A schematic flow diagram of the model on Aspen Plus is depicted in Fig. 2.

The model consists of mass and energy streams, and unit operations. The mass streams include the mass flow rates of air, biomass, and series of products that are limited to H_2_, CO, CO_2_, N_2_, H_2_O, CH_4_, H_2_S, and pure solid carbon (C). All the components except biomass and ash were modelled as conventional mixed streams using the chemical component library of Aspen Plus. The biomass and ash were modelled as non-conventional streams and were defined by using their ultimate and proximate compositions. The energy streams include the flow of energy from biomass, air and product streams. The energy content of the biomass was defined using the fuel’s lower heating value. The unit operations of the processes were modelled using the building blocks available in the software. The simulations were carried out at standard atmospheric pressure.

The air drying of the partially dewatered moist biomass was modelled using a stoichiometry-based reactor (RSTOIC) coupled to a flash separator. This RSTOIC reactor was used to define the conversion and vaporisation of some of the moisture in moist faeces while the flash separator separates the vapour from the solids. The gasifier was modelled by using three different units, a yield-based reactor (RYIELD), a Gibbs minimization-based reactor (RGIBBS) and a split block (SSPLIT). In a first stage, the stream of dried (partially or completely) faecal biomass was fed into the RYIELD reactor, which decomposes the dry biomass into individual constituents. The stream containing the elemental constituents was then introduced into the RGIBBS reactor, along with the heat from the decomposed biomass. The RGIBBS reactor was used for modelling the non-stoichiometric chemical equilibrium reactions that gives rise to heterogeneous mixtures of product gas. This block minimizes the Gibbs free energy under constraints of elemental balance and at defined temperature and pressure conditions, without considering the gasifier design. The air stream was also introduced into the RGIBBS reactor with prior flow through a heater. This heater preheats the air at a specified temperature (if required). The flow rate of the air stream was adjusted accordingly to enhance or reduce gasification. The separation of the gas products and the remaining solids (ash and unburned carbon) was modelled using SSPLIT.

The assumptions adopted in the model are as follows:

• Ideal gas behaviour for all gases.
• Air is dry and composed of 21 vol.% oxygen and 79 vol.% nitrogen.
• Steady state simulation.
• The reactions are adiabatic i.e. no heat loss.
• Product gas has sufficient time to reach chemical equilibrium.
• Carbon output consists and has molecular weight of pure carbon.
• The reaction is free of tar production.
• Steam is not introduced, except the water vapour released from the fuel moisture.
• No physical exergies are associated to air at standard temperature and pressure.
• Potential and kinetic exergies are negligible.

### 2.3 Fuel characterisation

Ultimate and proximate analyses of human faeces were carried out in order to gather data to feed the process model. Twelve (12) fresh human faeces were collected and characterised under the approved procedures of the Cranfield University Ethics System. Simulant faeces (SS) was prepared using the Recipe 9 in PRG [28] while wood biomass (WP) was obtained locally and used for comparison. All analyses were duplicated, and average values are reported. The proximate analysis was carried out using the methods outlined in BS EN 14774-3, BS EN 14775 and BS EN 15148 for moisture, ash and volatile matter determination respectively. For moisture content analysis, fresh samples were oven-dried to constant weight at 105 ± 5 °C in a convection hot air-oven (Heratherm Thermo Scientific OGS). The weight difference between the before- and after-oven-dried samples was used to deduce the relative moisture for each sample. The volatile matter and ash content were determined using a Carbolite muffle furnace with heating conditions described for solid biofuels. The fixed carbon content was obtained by subtracting the wt.% percent of moisture, ash and volatile matter from 100%. The ultimate composition was determined using the method outlined in BS EN 15104. The relative percentages of C, H, and N were determined using a thermal elemental analyser (Vario ELIII CHN). The oxygen content was obtained by subtracting the wt.% percent of C, H, N and ash from 100%, based on the method outlined in BS EN ISO 17247. The lower (LHV_BIOMASS_) and higher (HHV_BIOMASS_) heating value of the biomass were derived using (1), (2), (3), while the lower heating value of the gas (LHV_GAS_) was derived using Eq. (4).

(1) $${\text{HHV}}_{\text{BIOMASS}}\;(\text{MJ}/\text{kg})=0.3491\;[\text{C}]+1.1783\;[\text{H}]+{\left[0.1005\;[\text{S}]\right]}^{*}-0.1340\;[\text{O}]-0.151\;[\text{N}]-0.0211\;[\text{Ash}]$$

^∗^the term [0.1005 [S]]^∗^ was only considered for samples obtained from Ptasinski et al. [30] for model validation, since sulphur content was not determined in the faecal samples.

(2) $${\text{HHV}}_{\text{BIOMASS}}\;(\text{MJ}/\text{kmol})={\text{HHV}}_{\text{BIOMASS}}\;(\text{MJ}/\text{kg})\times \text{M}f$$

(3) $${\text{LHV}}_{\text{BIOMASS}}\;(\text{MJ}/\text{kmol})={\text{HHV}}_{\text{BIOMASS}}\;(\text{MJ}/\text{kmol})-9\times {\text{M}}_{\text{H}}\times h_{\mathrm{fg}}$$

(4) $${\text{LHV}}_{\text{GAS}}\;(\text{MJ}/\text{kmol})=241827\;(x_{{\text{H}}_{2}})+802303\;(x_{{\text{CH}}_{4}})+282993\;(x_{\text{CO}})$$

where C, H, O, N, S and Ash are respective weight percentages of mass of carbon, hydrogen, oxygen, nitrogen, sulphur and ash in the dry solid biomass fuel or sample. M*f* is the molecular mass of the biomass per kmol. *h_fg_* is the enthalpy of vaporisation of water (44.01 kJ/kmol at 25 °C). *x_i_* are the molar fractions of H_2_, CH_4_ and CO. M_H_ is the molecular mass of H_2_ per kmol.

### 2.4 Thermodynamic analysis

Energy and exergy analyses were carried out using the methods outlined in Ptasinski et al. [30], Karamarkovic and Karamarkovic [15] and Mhilu [22], as summarised in the Supplementary Information. Parameters such as equivalence ratio (ER), moisture content were varied to determine their influence on cold gas and exergy efficiencies. Prior to this analysis, model validation was carried out using the compositions and reaction conditions for coal, sludge, untreated wood, straw and manure, a data that was obtained from Ptasinski et al. [30] and Desrosiers [7]. The comparison between the model results and the reference data was carried out by determining the error margin via Root Mean Square (RMS)—Eq. (5).

(5) $${\text{RMS}}_{i}=\sqrt{\frac{\sum_{i}({\text{REF}}_{i}-{\text{MOD}}_{i})2}{D}}$$

where REF is the value obtained from other studies, MOD is the value obtained from the study model and *D* is the number of data for *i* species (H_2_, CO, CO_2_, H_2_, CH_4_, N_2_, H_2_S) (see Table 1).

## 3 Results and discussion

### 3.1 Model validation

The comparison of the compositions of the gas produced from the gasification of various samples (coal, sludge, untreated wood, straw and manure), as obtained from the current model and the thermodynamic model previously developed by Ptasinski et al. [30] is presented in Table 2.

**Fig. 3:**
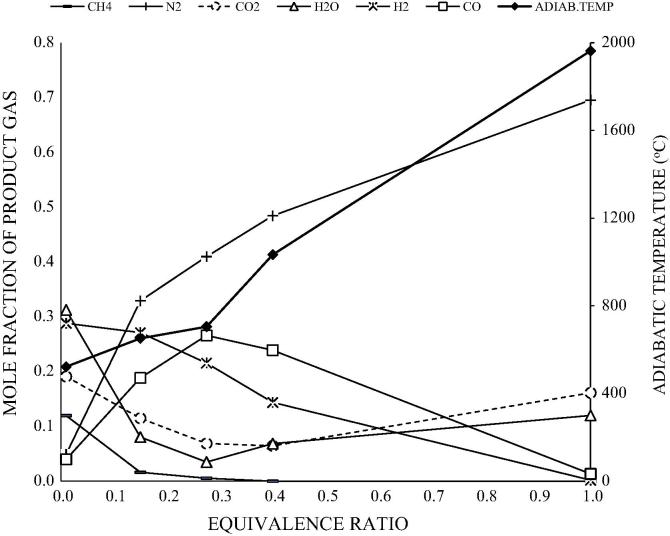
Molar fractions of product gas and adiabatic flame temperature as a function of equivalence ratio.

**Fig. 4:**
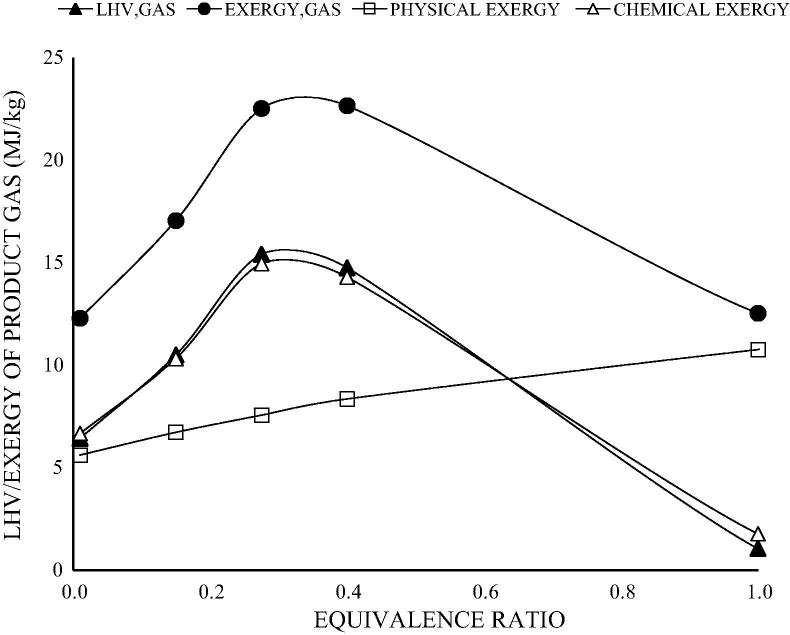
LHV and exergy of product gas as a function of equivalence ratio.

The results obtained from the model are satisfactorily close to the results reported in Ptasinski et al. [30], particularly in the case of coal. The RMS error for the coal sample varied from 0.0% to 0.2%. For the rest of the samples, hydrogen and carbon dioxide were under-predicted, while carbon monoxide and water were over-predicted. Nevertheless, the overall RMS error was less than 0.070 for all the components. The differences observed can be attributed to the choice of restricting the temperature approach for the equilibrium reactions, a common practise for modifying experimental results to simulation studies. This ensures that methane compositions are not over-predicted. The methane concentrations obtained in this model are indicative of the optimum conditions for gasification [30], [15] and validate the use of this model for further studies. The validated model was applied to a parametric analysis using dry ash free wood biomass across a range of equivalence ratio (pyrolysis, gasification and combustion), moisture content and pre-heated air temperatures. Results are shown in Fig. 3, Fig. 4.

Fig. 3 shows the adiabatic flame temperature (AFT) of the product gas at varying ER. The AFT at equivalence ratio of 0.01 corresponds to ∼522 °C. The addition of 1.75 kg air/kg of biomass (ER = 0.275) increases the temperature to ∼702 °C. Further addition of air up to 6.36 kg air/kg of biomass, the stoichiometric air flow rate (ER = 1), increases the AFT rapidly to 1967 °C. Further increases in the ER value result in a decline in temperature. The figure also shows an initial increase in CO concentrations with ER, achieving a maximum value at ER of 0.275, beyond which CO concentration declines. CH_4_ decreases with ER concentrations and becomes negligible after values of ER around 0.275. H_2_ concentration decreased with ER although at slower pace than CH_4_ concentration. The CO_2_ and H_2_O concentrations decreased to a minimum value at ER about 0.275 and moderately increase at higher values of ER. As expected, the N_2_ concentrations increased progressively with increasing ER values. The result trends shown in Fig. 3 are comparable with Desrosiers [7] considering similar fuel composition and gasification conditions.

From the above analysis, the ER of 0.275 is defined as the carbon boundary point (CBP) that is the point at which all the carbon in the biomass exists as CO, CO_2_ and CH_4_ [7], [29]. It is the optimum point at which syngas of the highest energy quality is produced. Beyond the CBP and as ER increases, there is progressive predominance of CO_2_ and H_2_O, because oxygen is in excess of the solid carbon. This oxidative exothermic conversion of the product gas leads to increasing reaction temperature.

Fig. 4 presents the outcomes of the energy and exergy analysis that further corroborates the results in Fig. 3. At the CBP, the gas attains energy and exergy values of 15 MJ/kg and 23 MJ/kg respectively with process efficiency of 74% (cold gas efficiency, CGE) and 95% (exergy efficiency, *η_ex_*), and limited exergy loss of 5%.

Further observations in Fig. 4 show that the subsequent addition of air beyond the CBP results in an increase in the sensible heat of the gas as ER progresses, but with consequential reduction in the chemical exergy of the product gas. The increase in sensible heat of the gas is however not sufficient to compensate for the reduction in chemical exergies, hence there is an overall reduction in exergy of the reaction beyond the CBP. The results also show that the chemical exergy across all equivalence ratios is lower than the energy of the product gas. This is because the chemical exergies of individual components are lower than their LHV counterparts. The outcome of the exergy analysis is more appropriate for the application targeted in this paper due to the potential recovery of thermal energy for drying the moist faecal biomass prior or during combustion. The sensible heat energy is excluded in the energy analysis.

**Fig. 5:**
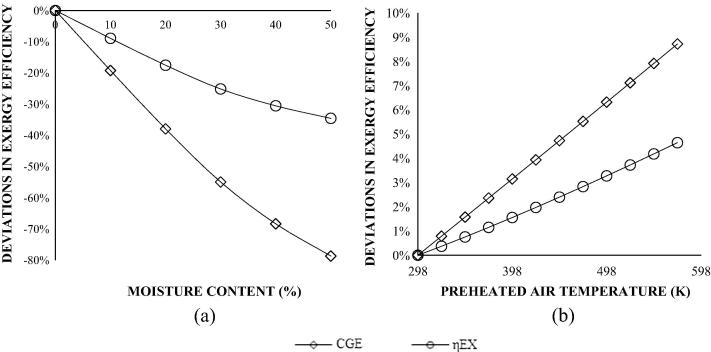
Deviations (%) in exergy efficiencies as a function of (a) moisture content, and (b) pre-heated air temperature.

Fig. 5a and b presents the influence of moisture content and pre-heated air temperature on cold gas and exergy efficiencies for adiabatic gasification of the dry wood biomass at fixed ER of 0.275 (CBP). The results are expressed as percentage deviations in exergy efficiencies.

Fig. 5a shows that a moisture content of 50% in the sample reduces cold gas energy and exergy efficiency by about 80% and 30% respectively. The absence of sensible heat estimation in the energy analysis accounts for the large variations in the CGE and exergy efficiency of the dry wood biomass. Fig. 5b shows the benefits of increasing the temperature of the gasifying medium, prior to introducing into the reactor. For every degree rise in preheated air temperature, there was a corresponding 0.02% improvement in *η_ex_* and 0.03% rise in CGE.

### 3.2 Human faeces as a potential biomass feedstock

Table 3 presents the proximate and ultimate compositions of the biomass collected in this study as wt.% as received basis (arb) and wt.% on dry-weight basis (db) respectively.

The results in Table 3 show that an average adult human faeces (AVGHF) approximately contain 77 ± 4 wt.% moisture, 12 ± 6 wt.% volatile matter, 7 ± 5 wt.% fixed carbon and 4 ± 1 wt.% ash, on as received basis, resulting in 51 ± 20 wt.% volatile matter, 32 ± 21 wt.% fixed carbon and 17 ± 1 wt.% ash, on a dry-weight basis. Based on the above analyses, it can be observed that the fixed carbon content of these samples had the highest variations, with some samples (HF1, HF3 and HF4) having little or no FC content, while samples (HF2, HF5-8) having FC content as high as 10–15 wt.% arb. Ash content had the least variations, values between 3 and 6 wt.% arb. Table 3 also presents the ultimate composition of an average adult human faeces as 51 ± 2 wt.% C, 7 ± 0 wt.% H, 4 ± 1 wt.% N, 21 ± 3 wt.% O and 17 ± 1 wt.% Ash, resulting in LHV of 21 ± 1 MJ/kg. Other constituents such as chlorine were not determined, hence, the oxygen composition that was obtained by subtracting the wt.% percent of C, H, N and ash from 100%, is an approximate estimate.

According to Feachem et al. [9] as reported by Rose et al. [32], nitrogen represents about 5–7 wt.% of human faeces on dry basis, a similar range to the values reported in this study. Nitrogen is said to be present in faeces in the form of undigested protein, nucleic acid, bacterial protein and intestinal shedding while lipid/fats account for ∼16 wt.% of the dry faeces. Afolabi et al. [1] showed that human faeces was composed of 37 wt.% C, 6 wt.% H, 5 wt.% N and 52 wt.% O, however there was no reference to the ash content and the basis for the calculation, therefore the oxygen composition might be lower. The moisture content and total solids in this study are within the range reported in Rose et al. [32], values of 63–86 wt.% arb and 17–34 wt.% arb in the same order. Wolley et al. [41] also obtained a mean moisture content of ∼77% with a standard deviation of ∼8%, which is similar to the value for the AVGHF. Afolabi et al. [1] however reported high values of ∼96 wt.% for moisture content and ∼4 wt.% for dry solids. Wolley et al*.* (2014) further reported values of ∼80–92% db for volatile matter, while Afolabi et al. [1] reported values of ∼71% and ∼29% for volatile matter and fixed carbon. These values are similar to the results in Table 3 for volatile matter. The differences in fixed carbon content could be due to a dry ash free basis calculation in Afolabi et al. [1], although this was not explicitly stated, while dry basis was used in this study. Torondel [37] stated that about 84 wt.% of dry solids of human faeces are organic matter, and largely constituted by bacterial biomass (up to 55 wt.%), and residual solid fibre accounts for the rest.

Proximate and ultimate analyses of wood biomass and simulant faeces were also performed. The percentages of C, H, N, O and ash on a dry-weight basis were 49 wt.%, 7 wt.%, 0.2 wt.%, 43 wt.% and 0.7 wt.% for wood biomass and 46 wt.%, 8 wt.%, 3 wt.%, 30 wt.% and 14 wt.% for simulant faeces. These values were similar to the AVGHF sample. Relating to the proximate composition, there were significant differences on as received basis, because the moisture and ash content in the wood biomass were 9 wt.% and 0.6 wt.% respectively, as opposed to the 77 wt.% and ∼11.7 wt.% in the AVGHF sample and in the same order. The proximate composition of the simulant faeces on the other hand was similar to the AVGHF sample, in terms of moisture and ash content, but insignificant quantities of fixed carbon and higher volatile matter content.

**Fig. 6:**
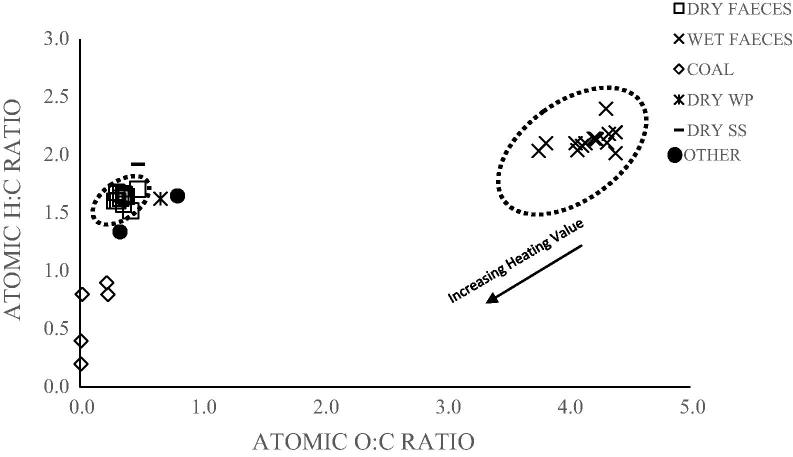
O/C and H/C atomic ratios of all human faeces samples (dry and moist) compared to other fuels.

To illustrate how faecal biomass (dry and moist) compare with other feedstocks such as coal, the elemental composition of all the human faeces, AVGHF, WP and SS samples were expressed as H/C and O/C atomic ratios. The ratios are represented in Fig. 6 using a Van Krevelen-type diagram [38].

As shown in Fig. 6, the moist human faeces sample have high O/C and H/C atomic ratios in the range of 3.5–4.5 and 0.25–0.47 respectively. These results were significantly higher than those of other samples including biomass and coal. In the Van Krevelen diagram, the range of O/C and H/C atomic ratios are 0.4–0.9 and 1.3–1.7 for other biomass and 0.0–0.2 and 0.2–0.9 for coal samples. Fig. 6 also indicates that the dry-weight faecal samples have O/C and H/C atomic ratios in the range of 1.5–1.7 and 2.2–2.4 respectively. These values were similar to other biomass samples but slightly higher to coal samples. The O/C and H/C atomic ratios for the dry human faeces show that the energy yield characteristic for the dry human faeces can be expected to be similar to other biomass samples, but lower for the moist faecal samples mainly because of the high O/C atomic ratios in the moist faecal samples. In this study, the LHV for wood biomass and simulant faeces were about 19 and 22 MJ/kg respectively while the LHV of the human faecal samples ranged from ∼19 to 22 MJ/kg (db).

Thus, the energy value for dry human faeces can be expected to be comparable to wood biomass and simulant faeces on dry-weight basis. The high moisture content in the fresh faecal samples can however affect the thermodynamic performance of the gasification process and reduce the total energy recovered. The gasification performance of dry and moist faeces were therefore assessed, as presented in Section 3.3.

### 3.3 Gasification of human faeces at the carbon boundary point

#### 3.3.1 Human faeces (dry-weight basis)

The validation analysis has demonstrated that the optimal operating conditions for gasification occur at the carbon boundary point. Although the sensible heat energy is higher under combustion equivalence ratios, the system’s total recoverable energy is considerable lower than at the CBP. Thus, this study focuses on evaluating the gasification of human faeces at the CBP operating conditions with the objective of recovering high energy gas products. Adiabatic gasification was performed using air over a range of equivalence ratios (0.01–1). The CBP temperature for each of the faecal samples (dry-weight basis) were determined, along with associated parameters and outputs such as air flow rates, product gas compositions, and energy and exergy values. The results are presented in Table 3 for the faecal samples, along with the outputs for AVGHF, WP and SS samples.

Table 4 shows that the product gas from the gasification of human faeces at the CBP varied in molar concentrations of H_2_, CO, CO_2_, and H_2_O, with values of 0.186–0.199, 0.217–0.233, 0.047–0.066 and 0.025–0.035 respectively. The molar concentrations of CH_4_ varied narrowly between 0.004 and 0.005 while N_2_ concentrations were in between 0.478 and 0.509. The CBP temperature had values in between 691 °C and 710 °C with corresponding air flow rates of 2.20–2.78 kg of air per kg of biomass. The ER for all the faecal samples were relatively the same at the CBP, values between 0.30 and 0.31. The gas resulting LHV values were within the range of 14–17 MJ/kg. The outputs from the exergy analysis present the physical exergy values of the dry faecal samples at about 7.0–7.5 MJ/kg and chemical exergy in the range of ∼14–16 MJ/kg. Thus, the resulting total exergy values were within the range of ∼21–24 MJ/kg. The total recoverable exergy value of the gas is however within the range of ∼19–22 MJ/kg, considering the energy and exergy required to dry the biomass to a moisture limit of 0%. Here, the enthalpy of vaporisation of water (44.01 kJ/kmol at 25 °C) and the energy to raise water from 25 °C to boiling point were removed from the total exergy of the gas. Faecal samples HF9 yielded product gas with the highest energy and exergy quality while HF2 gave rise to the gas product with the least energy and exergy quality. This could be attributed to the ultimate composition of the fuels mainly C and H. In this study, HF9 sample had a high C (∼50 wt.% db) and H (∼7 wt.% db) compositions and low O composition (∼20 wt.% db) while HF2 sample had low C (∼45 wt.% db) and H (∼6 wt.% db) compositions and the highest O composition (∼29 wt.% db). The AC was higher in HF9 (∼15 wt.% db) than HF2 (∼18 wt.% db) sample.

As can be seen in Table 4, the AVGHF sample yielded a gas product with slightly better energy content and higher CBP temperature than those from wood biomass and simulant faeces. This is because the compositions of C and H were higher for the AVGHF than the WP and SS. The AVGHF also had the lowest content in O among the three fuel samples, resulting in higher LHV of biomass and product gas. The SS used in this study can be considered as an appropriate simulant for human faeces, because it presented a similar energy yielding characteristics as the human faeces. The product gas from the simulant faeces had values of 681 °C, 2.38 kg of air per kg of biomass and 15.81 MJ/kg for CBP temperature, air flow rate and LHV at the CBP respectively. The LHV for SS sample compared well with samples HF4, HF8, and HF11, however, it was lower than the average faeces (AVGHF). A comparative exergy assessment of the outcomes for AVGHF, WP and SS samples shows that the product gas from AVGHF sample had slightly lower exergy yield than that of WP and SS. This is because the AVGHF sample had the least organic fractions and the highest ash composition among the three fuel types. Although, the chemical exergy of the gas for the AVGHF sample was higher at CBP than those of WP and SS samples, the sensible heat (physical exergy of the gas) was considerable lower for AVGHF. The process efficiencies for the adiabatic gasification of the dry human faeces varied from 70% to 100% for CGE and 90% to 100% for *η_ex_*). The CGE and *η_ex_* for the AVGHF sample were 70% and 92% respectively. In the case of the SS sample, the values for CGE and *η_ex,_* were 74% and 89% respectively. These efficiencies include the deductions of the energy and exergy requirement to dry the biomass to a moisture limit of 0 wt.%.

**Fig. 7:**
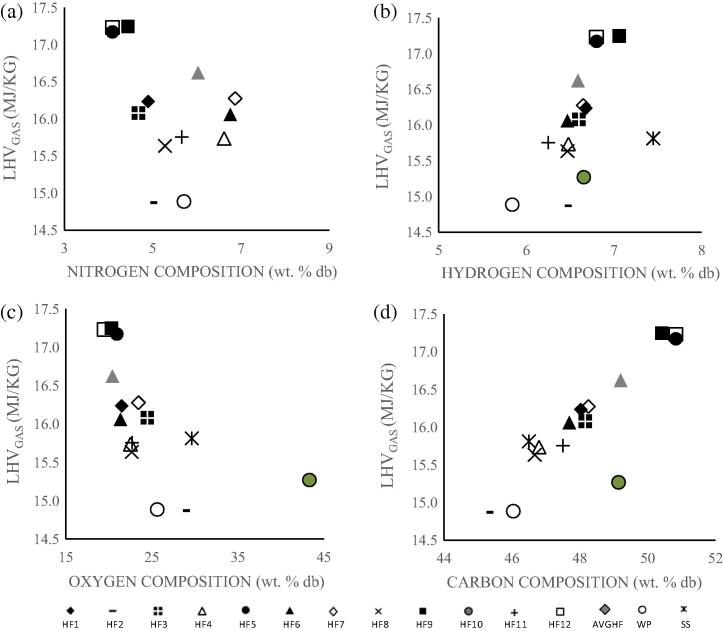
LHV of product gas as a function of wt.% db of (a) nitrogen, (b) hydrogen, (c) oxygen and (d) carbon composition.

To further illustrate the influence of fuel composition on gas energy value, the LHVs of the product gas from adiabatic gasification of dry human faeces at CBP were examined as a function of the percentages of C, H, N, O—Fig. 7a–d.

Fig. 7 shows that the high energy yielding dry human faeces with LHVs of at least 17 MJ/kg were HF12 and HF9. These samples contain a relatively high composition of C (>50 wt.% db) and H (∼7 wt.% db) while relatively low concentrations of O (<19 wt.% db) and N (<4.5 wt.% db). The lower energy yielding dry biomass were HF2 and HF10, both having LHVs of <15 MJ/kg. These samples conversely had a relative low C content (<45 wt.% db) and H content (<6 wt.% db.) The N and O compositions for these samples were relatively higher, with values above 5 wt.% db.

#### 3.3.2 Human faeces (as received basis)

The results in Section 3.3 showed that dry human faeces have high energy characteristics with slight variations in energy yield, even at optimum gasification operating conditions. In real systems, gasifiers designed for sanitary solutions would need to operate on fuels with varying fuel characteristics, in particular moisture and ash contents, and adapt operation to the varying fuel processed from the toilet units to maintain its performance. To this effect, this section examines the suitability of moist human faeces for energy conversion systems and their energy yield. The simulations were conducted for all the faecal samples (as received basis) using air across a range of equivalence ratios. The results are shown in Table 5 and includes a comparison with WP and SS samples for LHVs, CBP temperatures, air flow rates, product gas compositions, and energy and exergy values of the product gas at their optimum ERs.

Table 5 presents the shift in CBP with respect to fuel moisture content. Compared to the dry faecal samples, the range of equivalence ratios that was suitable for the conversion of these moist faecal samples (as received basis) increased to 0.55–0.62, a near combustion operating conditions. The energy of the resulting gas from the adiabatic gasification of these moist faeces reduced significantly to a range of 5.49–8.24 MJ/kg. These values were significantly lower to the LHV of wood biomass (WP) that contained 9% moisture with product gas energy yield of 14 MJ/kg. The reduction in LHV of the product gas is because part of the energy released from the conversion of biomass is used to drive the endothermic reactions of water vaporisation, such that only at higher equivalence ratios can the energy from the biomass conversion contribute to the energy of the product gas. It is only at these higher ERs that the carbon in the biomass exists as CO, CO_2_ and CH_4_ and reaches the CBP. For similar reasons, a reduced CBP temperature is observed in samples with higher moisture content, as reported in other studies [31], [30], [15].

Table 5 also shows that the product gas compositions also changed when considering the moist samples. The molar concentrations of H_2_, CO, CO_2_, and H_2_O were 0.062–0.085, 0.027–0.053, 0.0143–0.165 and 0.063–0.082 respectively. The molar concentrations of CH_4_ varied from 0.006 to 0.009 while N_2_ concentrations varied from 0.647 and 0.665. The CBP had values in between 504 °C and 550 °C with corresponding air flow rates of 4.42–5.18 kg of air per kg of biomass. HF10 remained the least energy yielded faecal sample while HF5 replaced HF12 and HF9 as the highest energy quality faecal sample. The product gas compositions changed under these conditions with a resulting poor energy yield gas because of the increasing oxidant concentration that consumes the product gas.

Despite the loss of quality in the product gas, the thermal energy of product gas increased and could be recovered to dry the biomass or for other useful purposes. This ensures that the process efficiencies are similar for both moist and dry faeces. The product gas had physical exergy values in the range of 9.27–13.86 MJ/kg and chemical exergy values of 5.85–8.41 MJ/kg, and the resulting total exergy were within the range of 19.43–21.85 MJ/kg. The physical exergies were higher than the chemical exergies, because of the increase in the sensible heat energy of the product gas, a result of increasing exothermic reactions. The process efficiencies varied from 39% to 47% for CGE while the *η_ex_* stayed relatively the same >90%. The CGE and *η_ex_* for the AVGHF, WP and SS samples were 45% and 100%, 65% and 92%, and 35% and 89% respectively. Comparing the results for the moist AVGHF, WP and SS to the dry counterparts, the results shows a significant reduction in LHVs by 53.1%, 6.7% and 52.4% respectively. The CBP temperatures also reduced by 17.1%, 1.9% and 16.9% while the air flow rates increased by 45.3%, 10.7%, and 46.3% in the same order. The moist SS was comparable to the faecal samples.

**Fig. 8:**
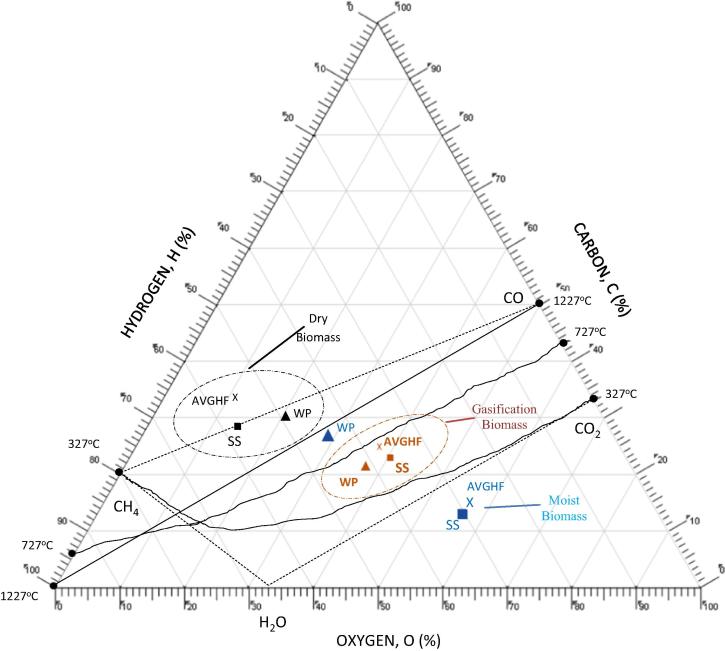
Molar CHO triangular diagram of dry and moist AVGHF, WP and SS prior to gasification and after gasification.

Since a gasifier is considered a CHON system [6], that is a system that operates on the atomic ratios of C, H, O, N, the carbon–hydrogen–oxygen (CHO) ternary diagram has been used to discuss the importance of operating the gasifier at optimum conditions and the influence of fuel moisture content. Studies by Tay et al. [36], Prins et al. [29], Cairns and Tevebaugh [5] have showed that CBP changes as a function of fuel characteristics, gasifying medium and operating conditions (pressure and temperature). The position of the biomass samples (as received and on dry basis) prior to gasification and after gasification at the CBP are denoted on the CHO ternary diagram – Fig. 8 using AVGHF, WP and SS samples. Nitrogen was not considered as it does not necessarily take part in the reaction, especially at combustion temperatures below 1557 °C. Cairns and Tevebaugh [5] have utilised a similar CHO system to establish the carbon deposition boundaries and gas phase equilibrium compositions for graphite at atmospheric pressure and at varying temperature range (25–1227 °C), and different O/H atomic ratios (0.026–2.5).

Fig. 8 shows four lines that connects CO, CH_4_, H_2_O, and CO_2_, referred to as the gasification zone in this study. Solid carbon is said to be present outside the connecting line for CO and CH_4_, free hydrogen is in excess beyond the line connecting CH_4_ and H_2_O, and O_2_ is in excess outside the connecting line of CO_2_ and H_2_O. The figure also presents carbon deposition boundary (CDB) lines for three different temperatures (327 °C, 727 °C and 1227 °C). As described in Tay et al. [36], Prins et al. [29], the solid line for a given temperature represents a carbon deposition boundary, in which all the equilibrium gaseous product does not co-exist with solid carbon. Below the carbon deposition boundary lines, no solid carbon is present, while above these lines solid carbon exists with gas phase equilibrium products. The carbon deposition boundary lines also consist of unique points on the CHO ternary system such that the equilibrium composition of H_2_, CO, CO_2_, H_2_O and, CH_4_ can be determined. Fig. 8 shows that the dry AVGHF, WP and SS samples are located around the CO–CH_4_ connecting line. The conversion of the dry AVGHF, WP and SS samples to gaseous products would require air or other gasifying medium in minimal amount to push this reaction towards a desirable gasification zone. Additional amount of air would be required for the gasification of the dry AVGHF. In the case of the moist samples, moist AVGHF and SS samples are outside the gasification zone and located in the zone of excess oxygen. This confirms that moisture is required to be removed before these reactions can proceed in the right direction. Fig. 8 further shows the position of the dry samples after adiabatic air gasification at the CBPs. Although, the CDB lines for each of the CBP temperature are not drawn, the figure illustrates that the products are located below the zone of carbon deposition, in this case (727 °C).

The high moisture content in human faeces is parasitic to gasification reactions, as it utilises part or whole of the heat generated to vaporise moisture. This could lead to delay ignition and subsequent conversion processes. It could also render the global energy balance of the system negative, since energy is required to pre-treat or dry the solids. Studies by Ptasinski et al. [30] have showed that there is a critical moisture limit at which gasification can occur. Above this critical moisture limit, a prior drying process of the moist biomass would be required. They compared the gasification efficiency of manure and sewage along with other carbon-based feedstocks including coal and identified a moisture limit requirement of 19 wt.% for the gasification of manure and sewage sludge the CBP temperature. Karamarkovic and Karamarkovic [15] examined this concept further by subjecting moist biomass to varying gasification temperatures and pressures. Their study showed that the removal of moisture from biomass improves energy and exergy efficiencies. They obtained the optimum energy and exergy efficiencies at moisture limit of 10 wt.%. Here, the CBP temperatures were 690 °C and 800 °C at pressures of 1 bar and 10 bar respectively. Their studies concluded that there is an optimum moisture limit for gasification at the CBP, beyond which there is decrease in energy and exergy efficiencies.

In this work, two modes for converting moist faecal biomass are suggested: (a) introducing the moist faeces into the gasifier at near combustion equivalence ratios, so that the heat released from the combustion reaction is used to vaporise the moisture or (b) drying of the faecal biomass to an allowable moisture limit prior to its introduction into the gasifier and aiming for CBP gasification. Irrespective of the route explored, there is an energy cost for drying the moist faecal biomass to an acceptable limit. The largest obstacle in this application is reducing the moisture content of faeces to a level that is suitable for gasification without an external heat source. In addition, an ultimate goal for the integrated Nano Membrane Toilet is that the energy recovered from gasification must be sufficient to fulfil the energy requirements of the system.

### 3.4 Influence of critical moisture limit at various equivalence ratio

In this study, the concept of the critical moisture limit is illustrated using the AVGHF sample. Conversion of the fully and partly-dried samples with varying moisture limits of 0–70 wt.% was evaluated at varying equivalence ratios (0.3–0.9). It was considered that no external heat source was supplied to the gasifier.

**Fig. 9:**
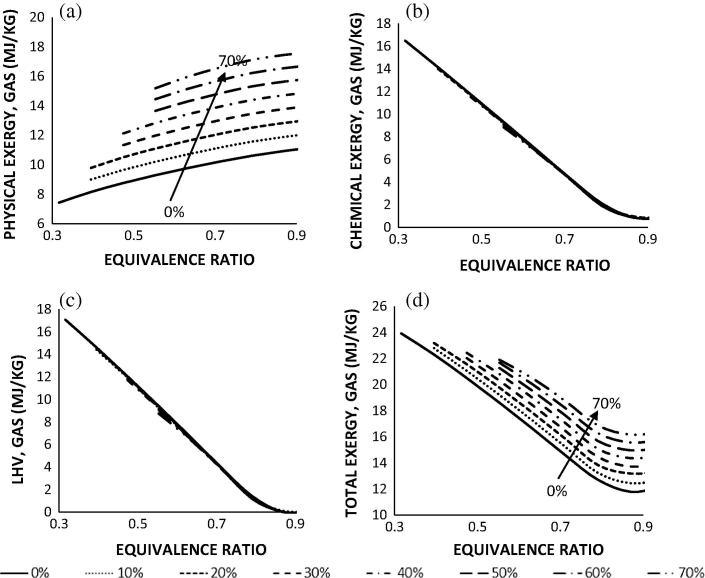
Influence of (a) physical exergy, (b) chemical exergy, (c) LHV, and (d) total exergy of the product gas for AVGHF sample as a function of equivalence ratio and moisture content.

Fig. 9a–d presents the physical exergy, chemical exergy, LHV and total exergy of the product gas as a function of ER and MC. Fig. 9a shows that the physical exergy of the gas increases with increasing moisture content of the sample and equivalence ratio. Thus, the physical exergy of the gas produced from the sample with 70 wt.% of moisture content is 15 MJ/kg at ER of 0.55 and increases up to 17 MJ/kg at ER of 0.90. These are higher values compared to the range of 9–11 MJ/kg for similar equivalence ratios in the dry sample. Fig. 9b and c shows that irrespective of the moisture content of the samples, the chemical exergy and LHV of the gas reduces with increasing ER as expected and explained in Section 3.1. These results depict the conversion of moist human faeces at and above the CBPs. Below the CBP, carbon is deposited, whereas beyond the CBP, the further addition of air results in the decrease of the LHV of the gas. This leads to low production of CO and H_2_, although CH_4_ concentrations increased slightly when compared to the gasification outputs of the dry faecal biomass. Karamarkovic and Karamarkovic [15] stated that the increase in CH_4_ concentration during the conversion of moist biomass is a result of a decrease in the reaction temperature at the CBP. The reduced CBP temperature increases the equilibrium constant for the methane forming reactions and more CH_4_ is formed as a result. However, the slight increase in the amount of CH_4_ concentrations does not compensate the loss of the LHV of the gas. These results are similarly reported in McKendry [21], Jarungthammachote and Dutta [14]. There was also increased molar concentrations of CO_2_ and H_2_O, and dilution of N_2_ concentrations in the product gas. The overall trends for the chemical exergy were similar as expected to the energy yield. The slight increase in physical exergy of the product gas did compensate for the overall improvement in exergy efficiencies. Fig. 9d depicts the final exergy of the product gas as decreasing with ER, however higher for samples with a moisture content higher than the dry sample. For instance, the total exergy of the gas was 19 MJ/kg at ER 0.55 and 12 MJ/kg at 0.90 for AVGHF (0%), but 22 MJ/kg at ER 0.55 and 16 MJ/kg at 0.90 for AVGHF (70%). As said above, this is because part of the energy removed from the system is available as sensible heat.

The analysis also shows that the acceptable moisture limit increases with increasing ER, i.e. ER of 0.32 (0 wt.%), 0.40 (10–20 wt.%), 0.47 (30–40 wt.%) and 0.55 (50–70 wt.%). These results imply that the optimum point at which the product gas has the highest energy quality for the dry human faecal samples occur at the CBP within gasification ER of about 0.3. However, with increasing moisture content in the faecal samples, the CBP changes to a higher ER and this consequently increases the acceptable moisture limit of the sample with slight loss in the exergy value of the gas. While the ER of 0.32 that corresponds to the CBP for the dry AVGHF sample cannot accommodate moisture in the sample, a shift to ER of 0.40 can accommodate up to 20% moisture in the sample, but at the cost of 3% loss of total exergy and 15% loss of LHV of the gas. Similarly, ER of 0.47 can accommodate up to 40% moisture, at the loss of 6% of the total exergy and 31% of the LHV of the gas while ER of 0.55 can accommodate up to 70%, at further costs of 8% and 49% in the same order. The progressive increase in the air flow requirements as the moisture content increases can be explained by the inefficient conversion of carbon in the moist biomass, hence a substantial amount of heat is required to evaporate the moisture.

The capacity of the gasifier to handle moist faeces and produce gas with high energy value is critical in the NMT application. To limit energy consumption, a mechanical process of dewatering will be most appropriate than a thermal treatment. In practice, this will depend on the existence, design and choice of pre-treatment method. To avoid the use of external heat, the above analyses show there is need for an appropriate choice of equivalence ratio for the level of moisture that would be expected in the gasifier, although this would be at a slight cost if the thermal heat and energy of the gas are recovered. Further difficulties might be encountered due to the physical, biological and chemical properties of faeces such as high moisture that is trapped within bacteria cells, a large constituent of human faeces.

**Fig. 10:**
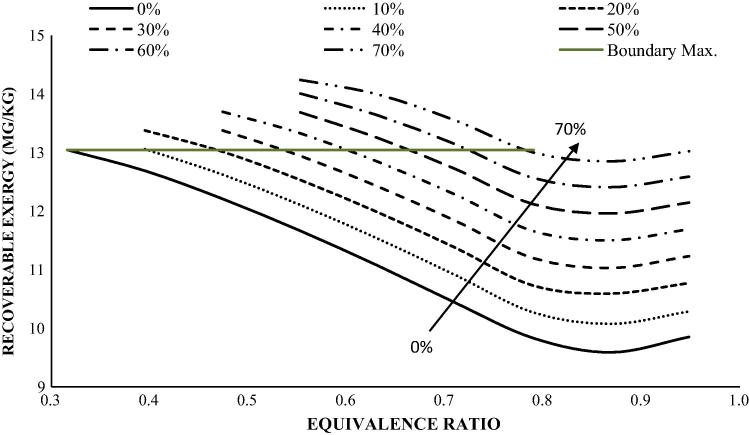
The potential recoverable energy for AVGHF sample as a function of equivalence ratio and moisture content.

The overall energy recovery will depend on the drier characteristics, method of heat transfer to the faeces (convective, conduction or radiative drying) and the efficiency of the heat recovery system. The rate of drying the fuel will also depends on a number of other factors such as surface area of the faeces, the relative humidity of the product gas, the temperature difference between the feed-in temperature of the faecal biomass and the hot gas leaving the gasifier and entering into the dryer, amongst other factors [8]. A large surface area for the faeces could significantly improve the rate of drying while large solid particle size could hinder the process. Considering a heat recovery efficiency of 80% as commonly reported for heat exchangers [4] and 40% thermal energy conversion of the heating value of the product gas for an indirect drying system [25], this study show that the recoverable exergy potential from an average samples of adult human faeces is within the range of 13–15 MJ/kg, provided the gasifier is operated at optimum conditions—Fig. 10. The results in Fig. 10 therefore shows that at, the acceptable moisture limit can be low as 10 wt.% for the moist AVGHF at adiabatic gasification ER of 0.4 and as much as 70 wt.% at adiabatic gasification ER of 0.79, beyond these points the total recoverable exergy potential will reduce beyond the optimum of the dry faeces. The analysis assumes that the energy required for drying the biomass is the amount of latent heat of evaporation supplied per kg of moisture removed. It excludes heat loss in the exhaust or from the body of the gasifier and drying units or as a result of distribution. It does not account for the energy consumption for dewatering the biomass or to break down the complex bonds in the faeces. Losses due to operational activities such as start-up/shut-down were not considered.

The average faeces generation rate for a healthy adult was reported as 110–170 g/cap/day [40], although human faeces generation capacities can vary as much as 51–796 g/cap/day [32], depending on dietary intake, fibre content, body weight and age. On this basis and considering a 77 wt.% of moisture in the faeces, as reported in this study, a self-sustained sanitary solution designed for a household of ten people should have the capacity to handle 0.1–1.7 kg of fresh faeces or 0.025–0.425 kg of dry faeces daily.

This study provides the upper limit on the product yields and energy recovery based selected on chemical species. It provides introductory insights on the suitability of human faeces as a feedstock for gasification and their energy recovery potentials for the development of an appropriate gasifier. It however excludes the production of tar that are also by-products of gasification of biomass. A pseudo-equilibrium model that takes into account some kinetic inputs and the energy requirements of the NMT is under development to support model validation with experimental results, and to enable a better understanding of the self-sustainability of the NMT.

## 4 Conclusion

The suitability of human faeces as a future energy source for self-sustained sanitary systems was investigated using thermodynamic analysis. The optimal routes for energy recovery were explored in view of fuel characteristics. The study concludes the following:

Human faeces were observed to contain high amount of moisture and significant quantities of ash, as much as 81.6 wt.% arb for MC and 5.6 wt.% arb for AC, when compared to wood biomass. The LHV of human faecal biomass was comparable or slightly better than wood biomass on a dry-weight basis. At the optimum CBP gasification ERs (0.30–0.31), the resulting energy and exergy values of the product gas were within the range of ∼15–17 MJ/kg and 22–24 MJ/kg respectively. This is because the compositions of carbon and hydrogen of a typical human faeces were higher and oxygen composition was lower than that of the wood biomass (WP) on a dry-weight basis. For suitable conversion of moist faecal samples, a near combustion operating condition would be required, if external energy source is not supplied, because part of the energy released during biomass conversion will be required to drive the endothermic processes of water vaporisation. At optimum near combustion ER (0.5–0.6), the product gas from moist faeces had reduced LHVs of 5–8 MJ/kg while the total exergy of the gas increased to a range of ∼19–22 MJ/kg. Comparing the dry and moist AVGHF, WP and SS samples, there was a reduction in the LHV of the gas by 53.1%, 6.7% and 52.4% and a corresponding increase in the air flow requirements by 45.3%, 10.7%, and 46.3% respectively. Furthermore, there was a reduction in CBP temperatures by 7.1%, 1.9% and 16.9% for the product gas recovered from moist AVGHF, WP and SS samples. At these operating conditions, the energy system can accommodate additional moisture in the sample at a slight loss of exergy values of the gas. Here, an ER of 0.40 improves the moisture limit to 20% but at the cost of 3% loss of total exergy, however a significant loss of the LHV of the gas (15% in this case). The study concludes that the recoverable exergy potential from an average adult moist human faeces is within the range of 13–15 MJ/KG.

## Figures and Tables

**Table 1:** Inputs of biomass feedstocks for model validation (data collected from Ptasinski et al. [30] and Desrosiers [7]).

Samples	Proximate analysis (wt.% arb)			Ultimate analysis (wt.% db)					LHV (MJ/kg db)
Moisture content	Ash content	Organic matter	C	H	N	O	S
Coal	11.5	8.5	80.0	78.5	5.0	13.3	1.5	1.7	24.84
Straw	12.7	6.4	80.9	49.0	6.0	44.0	0.8	0.2	14.62
Treated wood	19.8	1.8	78.4	51.4	6.0	41.3	1.2	0.1	15.29
Sludge	32.5	25.7	41.8	50.4	7.1	35.0	5.7	1.8	21.17
Manure	43.6	17.2	39.2	51.6	6.7	35.5	5.3	0.9	9.25
Wood biomass	–	–	100.0	52.5	6.2	0.0	0.1	41.2	8.43

arb-as received basis, db-dry basis.

**Table 2:** Data comparison between the model outputs and reference fuels in Ptasinski et al. [30].

Fuel type	Temp (°C)	Air flow (kg/kg_BIOMASS_)	H_2_ (mol%)	CO (mol%)	CO_2_ (mol%)	H_2_O (mol%)	CH_4_ (mol%)	N_2_ (mol%)	H_2_S (mol%)
Coal^a^	832	2.836	0.158	0.324	0.009	0.005	0.001	0.500	0.003
Coal^[MOD]^	832	2.836	0.158	0.327	0.008	0.005	0.000	0.499	0.003
Sludge^a^	600	1.237	0.192	0.056	0.147	0.186	0.004	0.412	0.003
Sludge^[MOD]^	600	1.237	0.113	0.151	0.056	0.270	0.000	0.408	0.003
Untreated wood^a^	642	1.452	0.227	0.177	0.126	0.076	0.013	0.380	0.000
Untreated wood^[MOD]^	642	1.452	0.224	0.231	0.079	0.094	0.000	0.371	0.000
Straw^a^	659	1.401	0.225	0.205	0.113	0.063	0.010	0.384	0.000
Straw^[MOD]^	659	1.401	0.195	0.266	0.055	0.107	0.000	0.376	0.000
Manure^a^	600	1.247	0.171	0.038	0.147	0.246	0.002	0.395	0.001
Manure^[MOD]^	600	1.247	0.104	0.117	0.072	0.313	0.000	0.392	0.001
RMS error (%)			0.048	0.066	0.062	0.053	0.008	0.006	0.000

a Data collected from literature.

**Table 3:** Proximate (wt.% arb) and ultimate (wt.% db) compositions of samples.

Samples	Proximate analysis (wt.% as received basis)				Ultimate analysis (wt.% dry basis)				LHV (MJ/kg dry basis)
Moisture content	Ash content	Volatile matter	Fixed carbon	Carbon	Hydrogen	Nitrogen	Oxygen^a^
HF1	70.50	5.60	23.90	0.00	48.03	6.68	4.90	21.49	20.84
HF2	79.20	3.10	6.90	10.80	45.25	6.44	4.95	28.61	19.06
HF3	74.50	4.10	21.30	0.10	48.16	6.60	4.68	24.46	20.57
HF4	78.20	3.80	18.00	0.00	46.80	6.48	6.62	22.50	20.14
HF5	69.80	5.40	11.30	13.50	49.20	6.59	6.03	20.40	21.26
HF6	77.00	4.10	8.20	10.70	47.69	6.47	6.76	21.34	20.54
HF7	78.30	3.20	8.50	10.00	48.25	6.65	6.87	23.44	20.76
HF8	77.90	4.20	7.90	10.00	46.67	6.47	5.28	22.65	20.06
HF9	81.60	3.30	5.40	9.70	50.42	7.06	4.44	20.29	22.16
HF10	80.40	3.30	8.80	7.50	46.04	5.84	5.71	25.61	18.95
HF11	75.20	4.40	11.70	8.70	47.50	6.25	5.66	22.68	20.12
HF12	81.60	3.50	8.90	6.00	50.83	6.80	4.09	19.43	22.10
AVGHF	77.00	11.70	4.00	7.30	50.83	6.80	4.09	20.91	20.56
WP	9.00	0.64	90.18	0.18	49.14	6.66	0.20	43.30	19.42
SS	77.59	3.11	19.27	0.03	46.50	7.45	2.57	29.64	20.45

HF-human faeces, WP-wood biomass, SS-simulant faeces, AVGHF-average composition of all human faeces samples.

a As 100 – (wt.% of C, H, N and ash).

**Table 4:** Adiabatic gasification of dry synthetic sludge, wood & faecal biomass at CBP.

Fuel type	EQ	Air flow (kg/kg_BIOMASS_)	CBP Temp. (K)	Molar concentrations (kmol%) db						LHV_GAS_ (MJ/kg)	εch,gas (MJ/kg)	εph,gas (MJ/kg)	Total exergy, gas (MJ/kg)
H_2_	CO	CO_2_	H_2_O	CH_4_	N_2_
HF1	0.30	2.58	975	0.193	0.221	0.052	0.028	0.004	0.502	16.23	15.72	7.26	22.98
HF2	0.31	2.22	964	0.199	0.217	0.066	0.035	0.005	0.478	14.87	14.43	7.30	21.73
HF3	0.31	2.48	972	0.194	0.224	0.055	0.029	0.005	0.493	16.08	15.57	7.39	22.96
HF4	0.31	2.46	976	0.193	0.221	0.053	0.029	0.004	0.499	15.73	15.24	7.12	22.36
HF5	0.31	2.64	982	0.189	0.227	0.048	0.025	0.004	0.506	16.62	16.08	7.15	23.23
HF6	0.31	2.53	980	0.191	0.225	0.050	0.027	0.004	0.503	16.06	15.54	7.08	22.62
HF7	0.31	2.50	975	0.194	0.224	0.053	0.028	0.004	0.497	16.27	15.76	7.27	23.03
HF8	0.30	2.46	973	0.193	0.220	0.055	0.029	0.004	0.498	15.63	15.14	7.17	22.31
HF9	0.31	2.77	979	0.194	0.221	0.049	0.027	0.004	0.505	17.25	16.69	7.46	24.15
HF10	0.30	2.26	976	0.186	0.233	0.057	0.028	0.004	0.491	14.88	14.41	7.02	21.43
HF11	0.31	2.46	978	0.188	0.229	0.052	0.027	0.004	0.500	15.75	15.25	7.08	22.33
HF12	0.31	2.78	983	0.189	0.227	0.047	0.025	0.004	0.509	17.23	16.66	7.31	23.97
AVGHF	0.31	2.73	980	0.190	0.228	0.048	0.025	0.004	0.505	17.17	16.61	7.39	24.00
WP	0.33	2.00	956	0.205	0.231	0.084	0.042	0.006	0.431	15.27	14.86	8.67	23.53
SS	0.31	2.38	954	0.210	0.201	0.069	0.041	0.006	0.472	15.81	15.37	8.08	23.45

HF-human faeces, WP-wood biomass, SS-simulant faeces, AVGHF-average composition of all human faeces samples.

**Table 5:** Adiabatic gasification of moist synthetic sludge, wood & faecal biomass at CBP.

Fuel type	EQ	Air flow (kg/kg_BIOMASS_)	CBP Temp. (K)	Molar concentrations (kmol%) db						LHV_GAS_ (MJ/kg)	εch,gas (MJ/kg)	εph,gas (MJ/kg)	Total εch,gas (MJ/kg)
H_2_	CO	CO_2_	H_2_O	CH_4_	N_2_
HF1	0.55	4.64	816	0.085	0.048	0.146	0.067	0.007	0.647	7.93	8.12	12.83	20.95
HF2	0.61	4.42	780	0.070	0.028	0.163	0.082	0.009	0.649	6.00	6.35	13.08	19.43
HF3	0.58	4.63	808	0.080	0.043	0.152	0.070	0.007	0.647	7.42	7.66	13.16	20.82
HF4	0.59	4.73	790	0.071	0.033	0.154	0.074	0.008	0.660	6.57	6.88	13.29	20.17
HF5	0.55	4.72	823	0.085	0.053	0.143	0.063	0.006	0.649	8.24	8.41	12.81	21.22
HF6	0.58	4.80	798	0.074	0.037	0.152	0.070	0.007	0.660	6.91	7.18	13.26	20.44
HF7	0.60	4.78	798	0.075	0.037	0.153	0.072	0.008	0.656	7.09	7.37	13.43	20.80
HF8	0.58	4.71	790	0.072	0.033	0.155	0.074	0.008	0.659	6.56	6.86	13.26	20.12
HF9	0.58	5.13	801	0.077	0.039	0.148	0.070	0.008	0.658	7.72	7.99	13.86	21.85
HF10	0.62	4.59	777	0.062	0.027	0.165	0.074	0.007	0.665	5.49	5.85	13.33	19.18
HF11	0.58	4.67	801	0.074	0.039	0.154	0.069	0.007	0.657	6.84	7.11	13.08	20.19
HF12	0.57	5.18	803	0.075	0.040	0.149	0.067	0.007	0.663	7.55	7.82	13.85	21.67
AVGHF	0.57	4.99	813	0.081	0.046	0.147	0.065	0.007	0.654	8.06	8.28	13.51	21.79
WP	0.37	2.24	938	0.187	0.201	0.099	0.048	0.006	0.459	14.31	13.98	9.27	23.25
SS	0.58	4.43	793	0.085	0.035	0.155	0.085	0.011	0.631	7.52	7.81	13.43	21.24

HF-human faeces, WP-wood biomass, SS-simulant faeces, AVGHF-average composition of all human faeces samples.
