# Supplementary material for: Energy recovery from human faeces via gasification: A thermodynamic equilibrium modelling approach
Source: Energy Convers Manag. 2016 Jun 15;118:364–76. doi: 10.1016/j.enconman.2016.04.005 (PMC4892428; doi:10.1016/j.enconman.2016.04.005)
Supplement: Supplementary data 1 [file mmc1.docx]

**Supplementary Information**

The method for thermodynamic analysis are well established in literature, however a summary is provided below indicating the processes for estimating the exergy of the biomass and gas. The gasifier performance was evaluated on the basis of exergy efficiency (η_ex_)_,_ assuming that potential and kinetic exergies are negligible and only chemical and physical exergies exist in the system —Eqn.1.

η_ex_ =$\frac{\text{ε}\text{product}}{\text{ε}\text{reactant}}\text{ }$_=_$\frac{\text{ ε}\text{ch, gas}\text{ + }\text{ε}\text{ph, gas}}{\text{ε}\text{ch, biomass}\text{ + }\text{ε}\text{ph, medium}}$ (Eqn.1)

where ε_ch, biomass_ is the chemical exergy of the biomass, ε_ch, gas_ is the chemical exergy of the product gas, ε_ph, biomass_ is the physical exergy of the gasifying medium and, ε_ph, gas_ is the physical exergy of the product gas. At standard temperature and pressure, ε_ph, medium_ is zero.

The chemical exergy of the gas, ε_ch_, _gas_ was determined using Eqns. 2 and 3:

$$\text{ε}\text{ch}\text{, gas }\text{= }\sum_{\text{i}} \text{J}\text{i }\text{ε}\text{ch, }\text{i}\text{ }\text{+}\text{ }\text{R}\text{o}\text{T}\text{o}\sum_{\text{i}} \text{J}\text{i }\text{ln}\text{ }\text{J}\text{i}\text{ }\text{ } \text{(Eqn}\text{.2}\text{)}$$

$$\text{J}\text{i}=\frac{\text{x}\text{i}\text{ }}{\sum\text{x}\text{i}} (Eqn.3)$$

where $\text{J}\text{i}$ and ε_ch,_ *i* are molar fractions of the product gas and the chemical exergy of the individual gas species, *i* respectively, R_o_ is the universal gas constant and T_o_ is the standard temperature (298.15K), *x*_i_ is the molar fractions of the product gas species, *i*. The values of ε_ch,_*_i_* are listed in Table 1 [Mhilu, 2012].

The chemical exergy of the biomass, ε_ch_, _BIOMASS_ was determined using Eqns. 4-6.

$\text{ε}\text{ch}\text{, biomass}\text{ = β LHV}\text{BIOMASS}$ (Eqn.4)

$\beta=\frac{\left[ \text{1.044 + 0.0160 Z}\text{H }\text{- 0.3493}\text{ }\text{Z}\text{O }\left[ \text{R} \right]\text{+ 0.0493 Z}\text{N} \right]}{\left[ \text{1-0.4124 Z}\text{O} \right]}$ (Eqn.5)

R = 1 + 0.0531 Z_H_  (Eqn.6)

LHV_BIOMASS_ remains the LHV of the biomass and β is the factor that is dependent on the hydrogen to carbon (H/C), oxygen to carbon (O/C), nitrogen to carbon (N/C) atomic ratios, signified as Z_H_, Z_O_, and Z_N_ in Eqns.7-9.

Z_H_ = H/C (Eqn.7)

Z_O_ = O/C (Eqn.8)

Z_N_ = N/C (Eqn.9)

The physical exergies, ε_ph, gas_ and ε_ph, air_ consider the sensible heat of the product gas and the gasifying medium. These were derived using Eqns. 10-11.

ε_ph, gas_ = (*h*_R_-*h*_0_) – T_0_ (*s*_R_-*s*_0_) (Eqn.10)

ε_ph, air_ = (*h*_H_-*h*_0_) – T_0_ (*s*_H_-*s*_0_) (Eqn.11)

where *h* and *s* are the molar specific enthalpy and molar specific entropy respectively and the subscript _“0”_ and _“R”_ describes the original state of the environment and the exit conditions for enthalpy and entropy of the product gas while _“R”_ describes the exit conditions for enthalpy and entropy of the gasifying medium, ideally the preheated air. All the enthalpy values (kJ/kmol) were obtained from the model outputs.

Cold gas energy efficiency, η_CGE_ was computed from the chemical energy of the gas in relation to the energy originally conserved in the biomass (Eqn. 12).

η_CGE_= $\frac{\text{LHV}\text{GAS}}{\text{LHV}\text{BIOMASS}}$ (Eqn.12)

Parameters such as equivalence ratio (ER), moisture content were varied to determine their influence on cold gas and exergy efficiencies. The ER was determined using Eqns.13-14

Equivalence Ratio (ER) = $\frac{\text{air flow rate }}{\text{fuel mass consumption rate x AFR}\text{STOIC}}$ $\text{(Eqn.1}\text{3}\text{)}$

AFR_STOIC_ = $\left( \frac{\text{M}\text{air}}{\text{M}\text{f}} \right)\text{STOIC}$ $\text{(}\text{Eqn}\text{.1}\text{4}\text{)}$

Table 1: Standard Chemical Exergy Values of Gaseous Components [Mhilu, 2012]

| Gaseous Species | $\text{ε}\text{ch}\text{, }\text{i}\text{ }\text{(kJ/}\text{kmol}\text{)}$ |
| --- | --- |
| H_2_ | 238490 |
| CO | 275430 |
| CO_2_ | 20140 |
| H_2_O (g$\text{)}$ | 11710 |
| CH_4_ | 836510 |
| N_2_ | 720 |
